# Effects of high concentrate rations on ruminal fermentation and microbiota of yaks

**DOI:** 10.3389/fmicb.2022.957152

**Published:** 2022-09-29

**Authors:** Kaiyue Pang, Dongwen Dai, Yingkui Yang, Xun Wang, Shujie Liu, Weihua Huang, Bin Xue, Shatuo Chai, ShuXiang Wang

**Affiliations:** ^1^Qinghai Academy of Animal Husbandry and Veterinary Sciences in Qinghai University, Xining, Qinghai, China; ^2^Key Laboratory of Plateau Grazing Animal Nutrition and Feed Science of Qinghai Province, Xining, Qinghai, China; ^3^Yak Engineering Technology Research Center of Qinghai Province, Xining, Qinghai, China

**Keywords:** high concentrate, rations, yak, rumen fermentation, rumen microorganisms

## Abstract

Ruminal microflora is closely correlated with the ruminant’s diet. However, information regarding the effect of high concentrate diets on rumen microflora in yaks is lacking. In the current study, 24 healthy male yaks were randomly assigned to two groups, each fed with different diets: less concentrate (LC; concentrate: coarse = 40: 60) and high concentrate (HC; concentrate: coarse = 80: 20) diets. Subsequently, a 21-day feeding trial was performed with the yaks, and rumen fluid samples were collected and compared using 16 s rRNA sequencing. The results showed that NH_3_-N, total VFA, acetate, butyrate, isobutyrate, and isovalerate were significantly higher in the HC group than that in the LC group (*p* < 0.05), while microbial diversity and richness were significantly lower in the HC group (*p* < 0.05). Principal coordinate analysis indicated that rumen microflora was significantly different in LC and HC groups (*p* < 0.05). In the rumen, phyla Firmicutes and Bacteroidota were the most abundant bacteria, with Firmicutes being more abundant, and Bacteroidota being less abundant in the HC group than those found in the LC group. *Christensenellaceae_R-7_group* and *Prevotella* are the highest abundant ones at the genus level. The relative abundance of *Acetitomaculum*, *Ruminococcus*, and *Candidatus_Saccharimonas* were significantly higher in the HC group than that in the LC group (*p* < 0.05), while the relative abundance of *Olsenella* was significantly lower in the HC group than in the LC group (*p* < 0.05). Compared to the LC group, the relative abundance of *Prevotella*, *Ruminococcus*, and *Candidatus_Saccharimonas* was significantly higher in the HC group. The relative abundances of *Prevotella*, *Prevotellaceae_UCG-003*, *Olsenella*, *Ruminococcus*, *Acetitomaculum*, *Candidatus_Saccharimonas*, and *NK4A214_group* were correlated with ruminal fermentation parameters (*p* < 0.05). Furthermore, PICRUSt 2 estimation indicated that microbial genes associated with valine, leucine, and isoleucine biosynthesis were overexpressed in the rumen microflora of yaks in the HC group (*p* < 0.05). Conclusively, our results suggest that high concentrate diets affect the microflora composition and fermentation function in yak rumen. The present findings would provide new insights into the health of yaks under high concentrate feeding conditions and serve as a potent reference for the short-term fattening processes of yaks.

## Introduction

Yak (*Bos grunniens*), an endemic livestock species living on the Qinghai-Tibetan Plateau (QTP), is an important means of production and livelihood for local herders (Long et al., 2018). In recent years, yak breeding has gradually developed from traditional grazing to a large-scale short-term fattening process, where high concentrate feeding is a common practice. The proportion of concentrate in the diet is an important factor affecting ruminant nutrition, and an appropriate proportion of concentrate can improve rumen microbiota (Petrir et al., 2012; Nugroho et al., 2013). When high concentrate diets are fed for a long period, the concentrate will be rapidly fermented in the rumen, producing large amounts of volatile fatty acids and lactic acid causing rumen acidosis as well as nutritional metabolic diseases. Severe metabolic diseases can induce mortality in ruminants, resulting in reduced economic efficiency (Malekkhahi et al., 2016; Nagata et al., 2018).

The rumen is the main site of digestion and metabolism in ruminants, and it contains microorganisms such as protozoa, bacteria, and fungi (Qiu et al., 2020). These microflora degrade diet fibers, produce volatile fatty acids, and use nitrogenous substances to synthesize microbial proteins that provide protein and energy to animals and are essential for the growth and reproduction of ruminant animals (Shabat et al., 2016). It is known that diet structure is an important factor affecting the composition of the rumen microbiota (Petri et al., 2013; Yáñez-Ruiz et al., 2015). The response of the dietary structure to ruminal microbiota has been a research focus in recent years. For example, the study by Fernando et al. (2010) used multiple molecular approaches to provide a more comprehensive illustration of the structure of the ruminal microbial community in beef cattle during adaptation from a high-grain diet to a forage diet. Mao et al. (2013) found that the effect of subacute rumen acidosis (SARA) adaptation on the rumen microbiota of dairy cows was studied by feeding a high-concentrate ration. However, to date, there is limited understanding of the effects of high concentrate diets on ruminal fermentation and ruminal microbiota, and their interactions. The current comprehensive analysis could provide valuable information to the short-term yak fattening industry.

We hypothesized that high concentrate feeds would affect ruminal fermentation, rumen fiber-degrading bacteria species, and rumen metabolic function of yaks, which in turn would affect the rumen health of yaks. As comprehensive analyses of the effects of high concentrate diets on yak microbiota are absent, in this study, we have used 16 s rRNA sequencing technology to analyze the effects of high concentrate rations on ruminal fermentation and rumen microbiota of yaks extensively and discussed the possible correlation of these two factors.

## Materials and methods

### Animals, diets, and experimental design

The feeding trial was conducted in September 2019 at the Lao Zhaxi breeding base, Guinan County, Qinghai Province, China. Twenty-four healthy male yaks at 3 weeks of age, with uniform health conditions (weight: 164.46 ± 31.18 kg), were randomly divided into two groups and each group was fed a full mixed ration of different diets: low concentrate (LC; concentrate: coarse = 40: 60) and high concentrate (HC; concentrate: coarse = 80: 20) diets. The diets were formulated according to the Chinese Beef Cattle Feeding Standard (NY/T815-2004), and the composition and nutrient contents of the two diets are shown in Table 1. Yaks were selected from grazing pastures. All yaks were uniformly numbered and fed alone. The rations were fed daily from 8: 00–9: 00 and 17:00–18:00 with free access to water. The pretest period was 7 days, and the experimental period was 21 days.

**Table 1:** Ingredients and nutritional composition of each diet.

Ingredients (%)	Group	
	LC	HC
Oat hay	60.00	20.00
Corn	3.75	44.09
Wheat bran	12.95	11.96
Rapeseed meal	11.00	11.92
Soybean meal	6.65	6.38
Palm oil powder^2)^	0.86	0.86
Rapeseed oil	0.80	0.80
NaCI	0.79	0.79
Premix^1^	3.20	3.20
Nutrient composition (%)		
DM	86.18	85.38
CP	15.43	15.82
EE	4.62	5.46
ME MJ/kg	9.40	11.53
NDF	45.58	25.60
ADF	27.24	13.80
Ca	0.29	0.23
P	0.44	0.48

The premix provided the following per kg of the diet: VA 3500 IU, VD 1000 IU, VE 40 IU, Mn 40 mg, Fe 50 mg, Cu 10 mg, Zn 40 mg, Se 0.3 mg; others were measured values.

### Sample collection and measurements

At the end of the experiment, before feeding in the morning, rumen fluid was collected using a bendable oral gastric tube with a metal filter, which was pre-cleaned by rinsing with clean warm water. The first 100 ml of rumen fluid was discarded to eliminate saliva contamination. Finally, a 50 ml rumen fluid sample from each yak was collected and filtered through four layers of gauze before measuring rumen pH with a pH meter (Model HI221, HANNA, Italy). The samples were divided into 15 ml sterile centrifuge tubes and stored in liquid nitrogen for the determination of ruminal fermentation parameters and microbiota analysis.

The filtered rumen fluid was centrifuged (at 17,000 *g* for 30 min at 4°C) to obtain the supernatant, which was further analyzed for NH_3_-N using phenol hypochlorite analysis (Broderick and Kang, 1980). Freshly prepared metaphosphoric acid (25% w/v, 2 ml) was added to filtered rumen fluid (8 ml) and then centrifuged (at 17,000 *g* for 10 min at 4°C). Volatile Fatty Acids (VFAs) concentrations were determined using gas chromatography (GC-2014; Shimadzu Corporation, Japan) as described by Cao et al. (2008).

### 16 s rRNA gene amplification and MiSeq sequencing

Microbial DNA was extracted using the CTAB (Sigma-Aldrich, Milan, Italy) method according to the instructions provided by the manufacturer. The purity and concentration of DNA were checked using 1% agarose gel electrophoresis. An appropriate amount of DNA sample was taken in a centrifuge tube and the sample was diluted to 1 ng/μL with sterile water. Specific primers with barcodes were synthesized for the V3-V4 variable region of the bacterial 16 s rRNA gene. Common primer sequences were as follows: 515F (5′-GTGCCAGCMGCCGCGG-3′) and 806R (5′-GGACTACHVGGGTWTCTAAT-3′). PCR (polymerase chain reaction) was performed using a 25 μl amplification system; 1 μl each of 5 μmol/l upstream and downstream primers and 5 ng of template DNA. Equal amounts of purified amplicons were pooled together to construct paired-end sequencing libraries, which were sequenced by Beijing Ovison Gene Technology Co., Ltd. (Beijing, China) using a platform (Mixed PE 300) according to a standard protocol.

#### Sequence and rumen microflora processing

The raw sequencing data were processed and filtered for quality using Trimmomatic (Version 0.36; Bolger et al., 2014) software, and valid sequences were obtained by removing chimeras through VSearch software and species databases. In addition, sequences with ≥97% similarity were categorized as operational taxonomic units (OTUs) using UPARSE software (*Uparse* v7.0.1001; Edgar, 2013).^1^ To obtain species classification information of each out, representative sequences were compared and analyzed using the RDP classifier algorithm version 2.2 (Wang, 2007) and Silvadatabase1 (Quast et al., 2012.), allowing community annotation at the kingdom, phylum, class, order, family, and genus levels. Alpha Diversity (Chao1, Shannon, PD-whole-tree, and Observed-species) was calculated by QIIME 2 (version 1.9.0), and the richness of the community was analyzed using Chao1 richness The Chao1 richness index (Chao1), the Shannon index, the coverage index of PD-whole-tree were applied to analyze the diversity of the community. The number of OTUs was analyzed using the observed-species index. Beta diversity was calculated based on the unweighted UniFracdistance and visualized by principal coordinate analysis (PCoA). Linear discriminant analysis effect sizes (LEfSe, LDA > 3) were used to identify important bacteria in both groups (Miller et al., 2016). To predict microbiota function and explore differences between the two groups, PICRUSt 2 software was used (Douglas et al., 2019).

### Statistical analysis

Independent samples t-test based on SAS (SAS, version 9.2) was applied to compare ruminal fermentation parameters between LC and HC groups, and the differences were considered statistically significant at *p* < 0.05. Microbial networks were generated using Gephi software (version 0.9.2)^2^ to calculate correlations between dominant taxa. Pearson correlation coefficients between the relative abundances of rumen bacteria (genera) and ruminal fermentation parameters were calculated using the heat map package in R software (version 4.0.2). Functional prediction of rumen microflora in yaks of LC and HC groups was studied with PICRUSt 2 and differences between the two groups in levels 1, 2, and 3 of the KEGG (Kyoto Encyclopedia of Genes and Genomes) pathway were determined.

## Results

### Ruminal fermentation parameters

The effect of high concentrate diets on ruminal fermentation parameters in yaks is shown in Table 2. NH_3_-N (*p* = 0.014), total VFA (*p* < 0.001), butyrate (*p* < 0.001), isobutyrate (*p* = 0.007), isovalerate (*p* < 0.001) were significantly higher in the HC group than those in the LC group (*p* < 0.05). The proportion of Acetate is higher in the LC group and the Propionate higher in the HC group.

**Table 2:** Effect of high concentrate diets on ruminal fermentation parameters in yaks.

Parameters	Treatment groups		SEM	*p*-Value
	LC	HC		
pH	6.42	6.13	0.06	0.074
MCP	2.21	2.85	0.43	0.273
NH_3_-N, mg/dL	16.76^b^	21.39^a^	0.86	0.014
TVFA, mM	60.93^b^	74.96^a^	1.95	<0.001
VFAs, molar % of TVFA				
Acetate	63.53^a^	61.18^b^	0.59	0.048
Propionate	22.69	22.78	1.09	0.928
Butyrate	10.68^b^	12.73^a^	0.40	<0.001
Isobutyrate	0.78^b^	0.91^a^	0.05	0.128
Valerate	1.52	1.23	0.11	0.077
Isovalerate	0.81^b^	1.17^a^	0.04	<0.001
Acetate: Propionate	2.88	2.72	0.09	0.366

MCP, microbial protein; NH_3_-N, ammonia nitrogen; TVFA, total volatile fatty acids; SEM, standard error of the mean. LC, low-concentrate group; HC, high-concentrate group.

a,b The values in same row with differenct superscript letter differ significantly (*p* < 0.05).

### Richness, diversity estimates, and rumen bacteria composition

**Figure 1:**
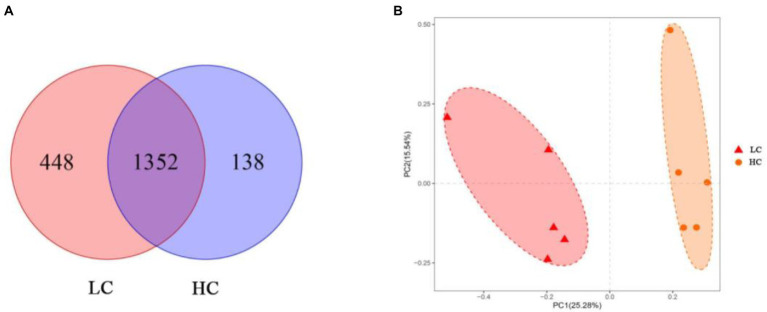
Differences in ruminal microbial communities and operational taxonomic units (OTUs) of yaks fed low concentrate (LC) and high concentrate diets (HC). Venn diagrams **(A)** show the specific and shared OTU between the two groups. Differences in rumen microflora between the two groups were calculated using weighted UniFrac distances **(B)** and coordinates were calculated using principal coordinate analysis (PCoA).

**Figure 2:**
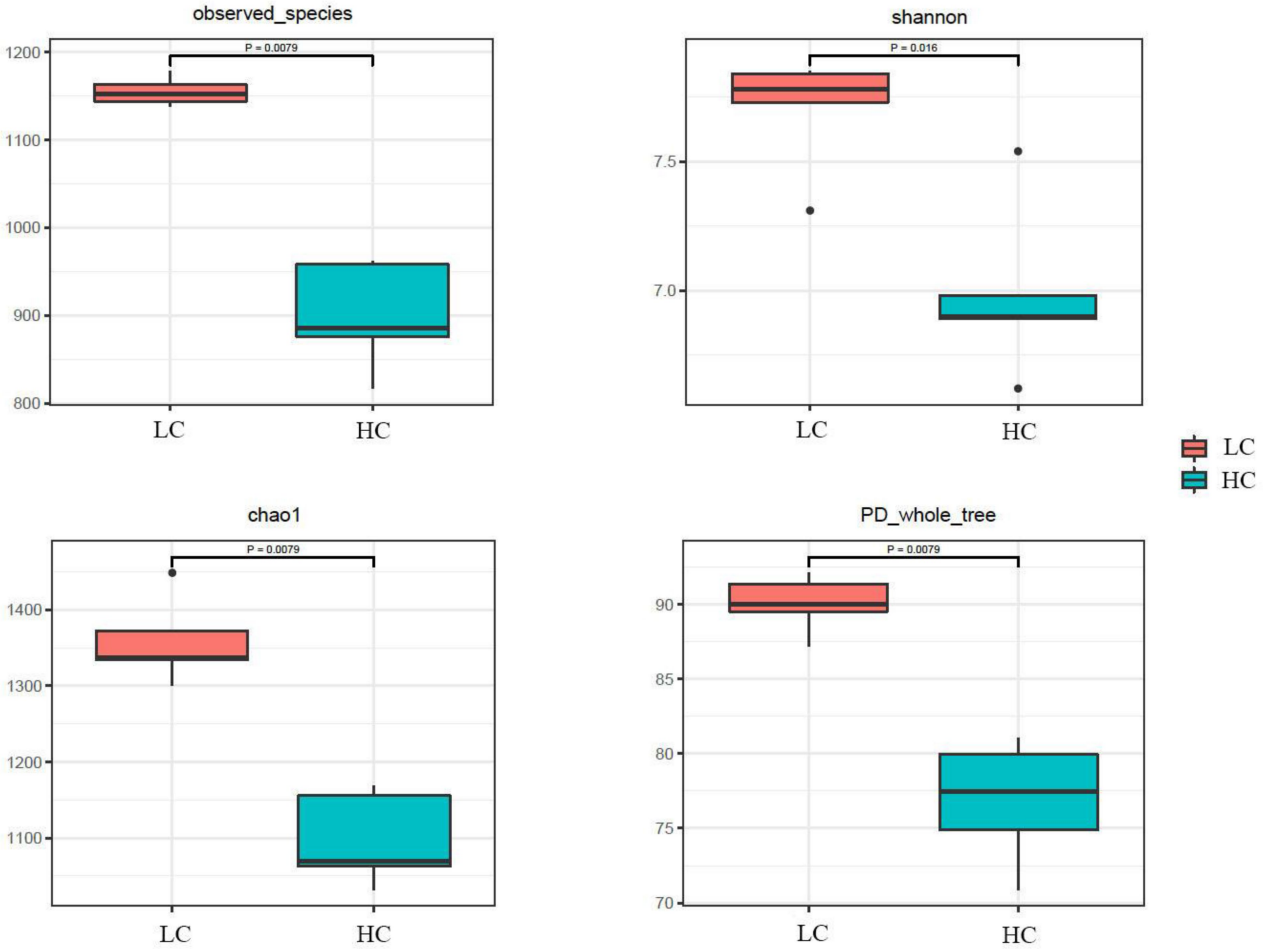
Diversity in the ruminal microbial community of yaks fed low concentrate (LC) and high concentrate diets (HC). *p* < 0.05 indicates a significant difference.

According to the Venn diagram, 1,352 OTUs were present in the rumen of yaks in LC and HC groups, with 448 and 138 unique OTUs, respectively (Figure 1A). According to PCoA (Figure 1B), significant differences were observed between the rumen microflora of the LC and HC groups. Alpha diversity calculations (Figure 2) showed significant differences in Chao1, Shannon, PD-whole-tree, and Observed-species between the two groups, indicating that Chao1, Shannon, PD-whole-tree, and Observed-species were significantly lower in the HC group than that in the LC group (*p* < 0.05).

### Bacterial compositions among treatment groups

**Figure 3:**
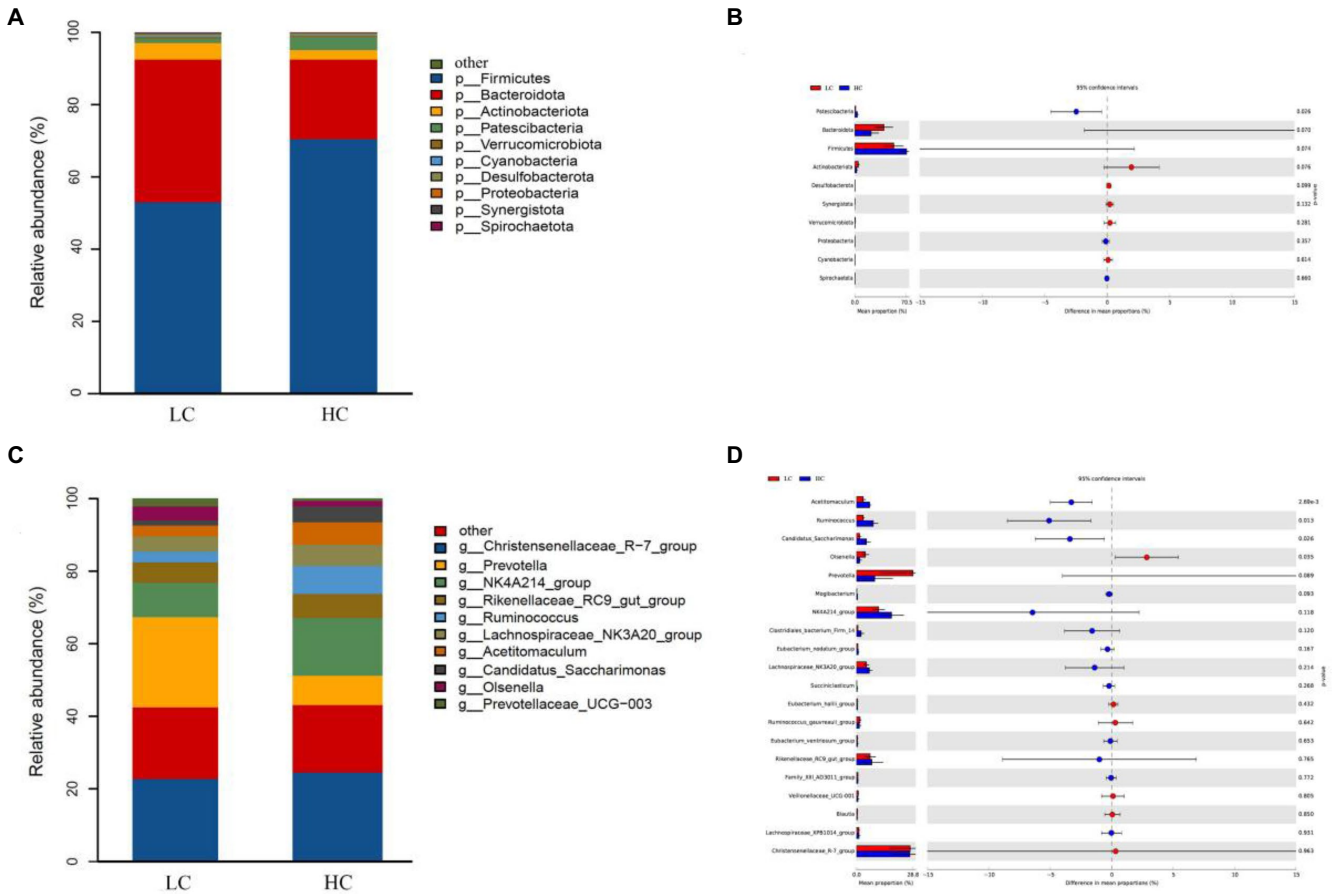
Classification of bacterial community composition in yaks fed low concentrate (LC) and high concentrate (HC) diets. **(A)** Phylum level. **(C)** Extended error bars showing the bacteria that differed between the two groups at the phylum level. **(B)** Genus level. **(D)** Extended error bars showing the two groups of bacteria that differed at the genus level.

We performed a taxonomic analysis of the 10 bacterial phyla identified. At the phylum level, among them, Firmicutes and Bacteroidota represented 61.55 and 30.69% of the total reads, respectively, followed by Actinobacteriota (3.65%), Patescibacteria (2.33%) and Verrucomicrobiota (0.46%; Figure 3A). The relative abundances of Patescibacteria and Firmicutes were higher in the HC group than that in the LC group, while a lower relative abundances of Bacteroidota, Actinobacteriota and Verrucomicrobiota were detected in the HC group (Figure 3B). A total of 167 genera was identified from rumen samples. *Christensenellaceae_R-7_group* (19.06%) and *Prevotella* (13.35%) were the most dominant genera, followed by *NK4A214_group* (10.17%), *Rikenellaceae_RC9_ gut_group* (4.97%), *Ruminococcus* (4.30%), *Lachnospiraceae_NK3A20_group* (4.11%), *Acetitomaculum* (3.60%), *Candidatus_Saccharimonas* (2.33%), *Olsenella* (2.18%), and *Prevotellaceae_UCG-003* (1.11%; Figure 3C). Among them, the relative abundance of *Acetitomaculum*, *Ruminococcus*, and *Candidatus_Saccharimonas* were significantly higher in the HC group than that in the LC group (*p* < 0.05), whereas the relative abundance of *Olsenella* was significantly lower in the HC group than that in the LC group (*p* < 0.05; Figure 3D).

**Figure 4:**
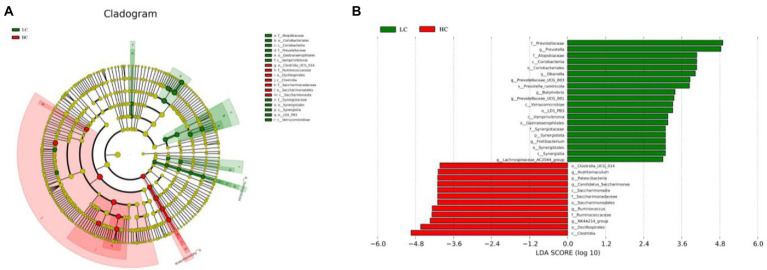
LEfSe analysis of rumen microflora of yaks fed low concentrate (LC) and high concentrate diets (HC). **(A)** Histogram of linear discriminant analysis scores based on categorical information. **(B)** Linear discriminant analysis effect size classification plot based on categorical information.

To better understand the dominance of specific bacteria in HC and LC groups, we used the LEfSe method (Figure 4). Figure 4A depicts a representative cladogram of the predominant microbiome structure, showing the most remarkable differences in taxa in the two groups. The data comparing the two groups indicated that 11 clades were more abundant in the LC group and seven clades were more abundant in the HC group. Prevotellaceae, *Prevotella*, Atopobiaceae, Coriobacteriia, Coriobacteriales, *Olsenella*, *Prevotellaceae _UCG-003*, and *Prevotella_ruminicola* were abundant in the LC group. *Clostridia_UCG_014*, Patescibacteria, *Candidatus_Saccharimonas*, Saccharimonadia, Saccharimonadaceae, Saccharimonadales, *Acetitomaculum*, *Ruminococcus*, *Ruminococcaceae*, *NK4A214_group*, Oscillospirales, and *Clostridia* were in the HC group in an Overrepresentation (Figure 4B).

### Network analysis of bacterial communities

**Figure 5:**
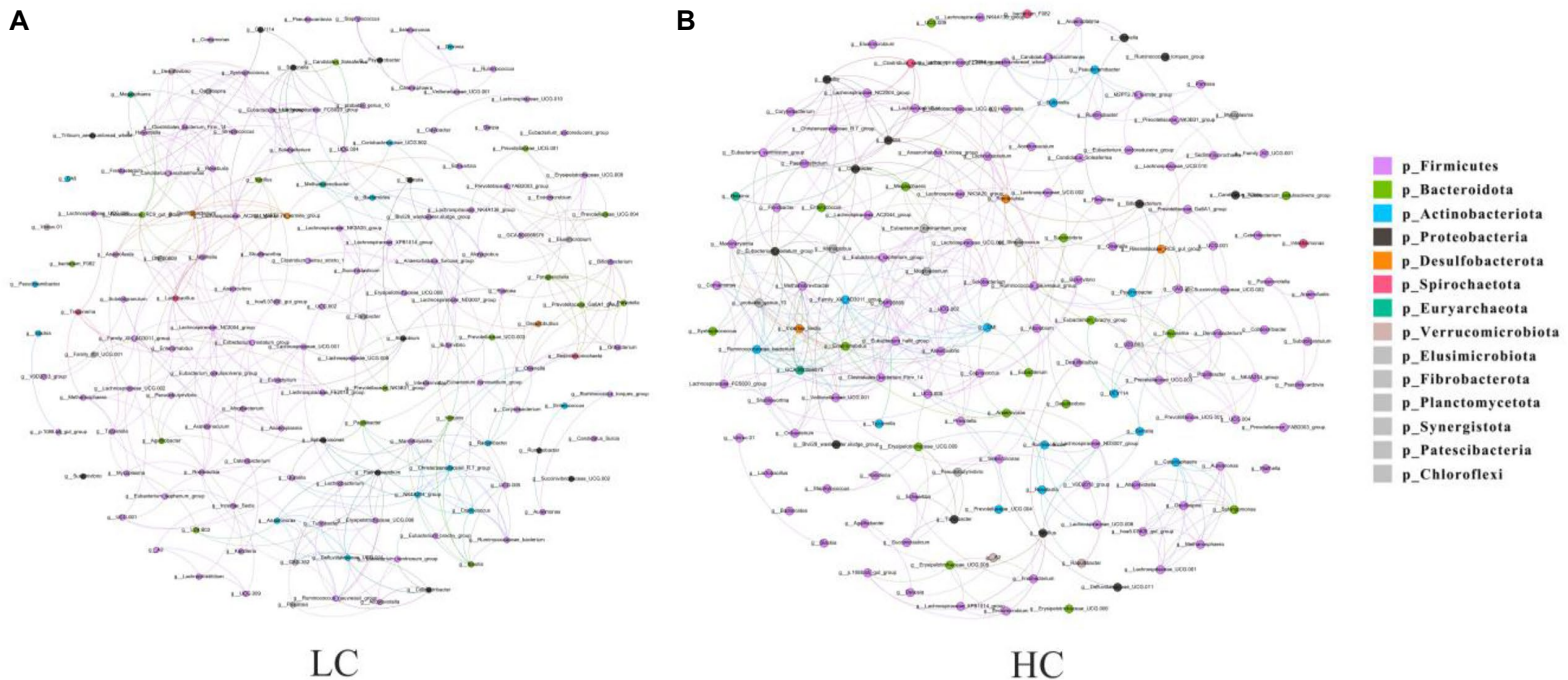
Interaction network of ruminal microflora. The ruminal microflora correlation network based on 16S rRNA genes showed statistically significant interactions with absolute values of correlation coefficients >0.6. The size of the nodes is scaled according to the abundance of each taxon in the microflora. The red line indicates a positive correlation and the green line indicates a negative correlation.

Microbial interactions between rumen bacterial communities in yaks were analyzed using microbial networks. The results showed that the high concentrate diet altered the correlation within the microbiota (Figure 5), and we verified that the negative correlation was stronger in the HC group than that observed in the LC group.

### Correlations between rumen bacteria and ruminal fermentation parameters

**Figure 6:**
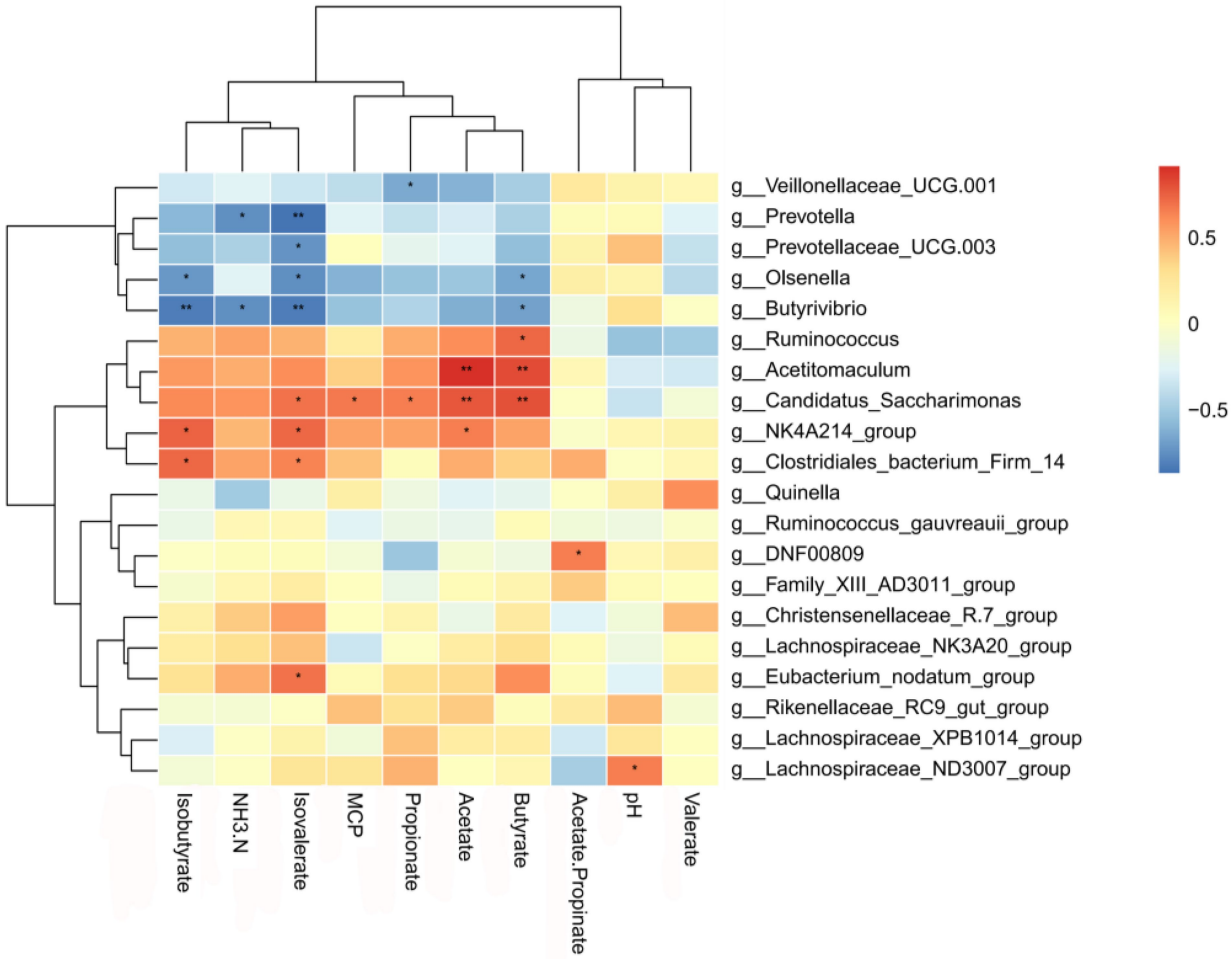
Correlation between bacteria and fermentation parameters in the rumen. Each row in the figure represents a genus, each column represents a metabolite, and each grid represents the Pearson correlation coefficient between a component and a metabolite. The red color represents a positive correlation, while the blue color represents a negative correlation. * and ** indicate significant levels of 0.05 and 0.01, respectively.

Correlation analyses between rumen bacteria and ruminal fermentation parameters were based on Spearman’s correlation coefficients, and significantly influential rumen microflora (genus level) were significantly correlated with ruminal fermentation parameters (Figure 6). *Prevotella* was negatively correlated with NH_3_-N and isovalerate concentrations; *Prevotellaceae_UCG-003* was negatively correlated with isovalerate; *Olsenella* was negatively correlated with Isobutyrate, isovalerate, and butyrate concentrations; *Ruminococcus* was positively correlated with Butyrate concentration; *Acetitomaculum* was positively correlated with acetate and butyrate concentrations. Positively correlation was observed for *Candidatus_Saccharimonas* with concentrations of sovalerate, MCP, propionate, acetate, and butyrate, and for *NK4A214_group* with isobutyrate, isovalerate, and acetate concentrations.

### PICRUSt2 function prediction

**Figure 7:**
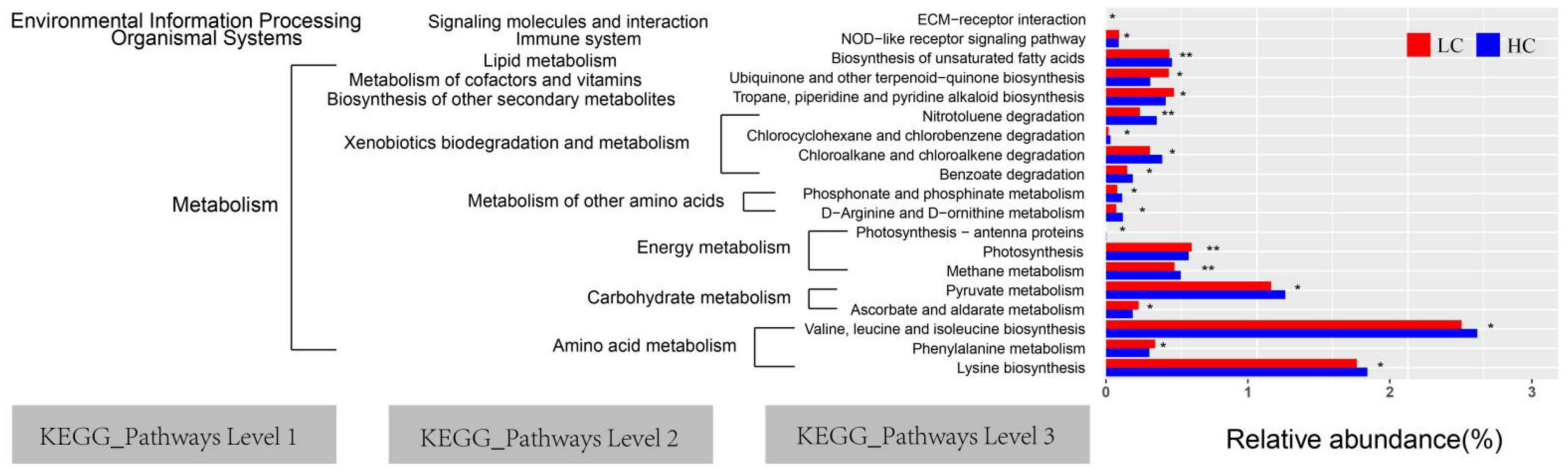
Yaks, which were fed low concentrate (LC) and high concentrate diets (HC), had significantly different functional predictions of the rumen microbiota of the KEGG pathway (*p <* 0.05). The graph shows the KEGG pathways for levels 1, 2, and 3. “*” and “**” indicate significance levels of 0.05 and 0.01, respectively.

PICRUSt 2 gene function assessment was used to predict the function of rumen microflora in LC and HC groups of yaks. The highest abundance of valine, leucine, and isoleucine biosyntheses (26.58%) was observed, followed by lysine biosynthesis (18.74%). PICRUSt 2 prediction software enriched 40 major pathways (relative abundance >1%) in the 3-level KEGG pathway, 19 of which showed significant differences between the LC and HC groups (*p* < 0.05; Figure 7). Notably, the relative abundance of metabolism of other amino acids and xenobiotics biodegradation were significantly increased in the HC group (*p* < 0.05).

## Discussion

In this study, we investigated the effects of high concentrate diet on microflora and fermentation in rumen of yaks and interactions between these two factors. A stable intra-rumen environment is particularly important for ruminants; ruminal pH, NH_3_-N and VFA molar concentrations are important indicators of a stable intra-rumen environment and reflect the status of ruminal fermentation (Tomczak et al., 2019). A stable ruminal pH is a prerequisite for its normal function, and it is determined by volatile fatty acid and lactic acid accumulation (Tomkins et al., 2015). The present results showed that the pH decreasing tendency in the HC group. The study conducted by Ogata et al. (2019) found that ruminal pH was significantly lower in Japanese black wagyu cattle that were fed a high grain diet. Similar results were obtained in other studies (Kmicikewycz et al., 2015; Ramos et al., 2021). The reduction in pH may have resulted from the significant change in the ruminal bacterial community as a result of feeding high concentrates. The increased frequency of lactic acid producing bacteria and lactic acid utilizing bacteria in the rumen and an imbalance in the bacterial flora leads to the accumulation of lactic acid in the rumen, further reducing ruminal pH. Moreover, the number of fiber degrading bacteria also decreases drastically due to the low fiber content in the diet and the accumulation of lactic acid in the rumen (Mackie and Gilchrist, 1979; Tajima et al., 2001; Lee et al., 2019).

NH_3_-N is endogenous ruminal nitrogen, a product of fermentation and decomposition of diet proteins, and a major component for microbial synthesis of bacteriophage proteins in the rumen (Garcia-Gonzalez et al., 2010; Thao et al., 2014; Lv et al., 2020). Carlos et al., 2021 reported that the NH_3_-N concentration in the rumen of dairy goats that were fed high concentrate diets was significantly higher than that of the low concentrate diet group. NH_3_-N concentrations in the current HC group were comparable to the above results and with those of Jahan et al. (2018); a large amount of nitrogenous substances in the high concentrate diet may be the possible reason for this outcome. The more nutrients in the rumen are decomposed by ruminal microorganisms to produce a larger amount of NH_3_-N, increasing NH_3_-N concentration and even ammonia toxicity in severe cases (Vcdsa et al. 2020).

Volatile fatty acids are derived from the fermentation of carbohydrates and proteins in the diet by rumen microorganisms and are an important source of energy for ruminants (Li et al., 2016). Acetic acid, propionic acid, and butyric acid in the rumen are the main components of volatile fatty acids (Guilloteau et al., 2010). Acetic and propionic acids enter the portal circulation and are metabolized in the liver (Minamoto et al., 2019), acetic acid is the main synthetic precursor of fat in ruminants (Hou et al., 2020), and propionic acid is converted to glucose by gluconeogenesis or enters the tricarboxylic acid cycle for oxidative energy supply (Young, 1977; Jia et al., 2018), and butyric acid is an important source of energy for animals (Gorka et al., 2009); in this study, the concentrations of Total VFA, Acetate, Propionate and Butyrate were higher in the HC group than in the LC group, which is similar to Bevans et al. (2005), Sato (2016), and Nagata et al. (2018) studies were similar. Due to the high percentage of concentrate in the diet, the diet enters the rumen to produce a large amount of VFA (including acetic acid, propionic acid and butyric acid) by rapid fermentation, resulting in a lower absorption rate of VFA and excessive accumulation of VFA in the rumen (Goad et al., 1998), which affects ruminal fermentation in yaks, which in turn can lead to reduced feed intake as well as digestive problems affecting the health of yaks.

We further investigated the effect of high concentrate diets on rumen microflora of yaks using 16S rDNA high-throughput sequencing technology. In the present study, both alpha and beta diversity indices of microflora were significantly different between the two groups, indicating that the diversity of rumen microflora in yaks was closely related to the diet concentrate ratio. Our results showed that intra-ruminal bacterial diversity and richness were reduced in yaks fed a high-concentrate diet, which was comparable with the results obtained by Petri et al. (2013) and Zened et al. (2013). This suggests that feeding high concentrate diets to yaks causes a decrease in rumen pH and inhibition of cellulose-degrading bacteria, leading to a decrease in the diversity and abundance of ruminal bacteria. PCoA analysis showed that rumen microbial communities aggregated according to the concentrate ratio in diets, and significant differences between intra-ruminal microbial communities were observed, which were consistent with previous reports (Hu et al., 2019; Islam et al., 2021).

In the current study, as similar to previous studies, Bacteroidota and Firmicutes were the dominant phyla among ruminal microorganisms in yaks (Zhou et al., 2017; Bi et al., 2018; Liu C. et al., 2019a; Fan et al., 2020), indicating that these bacteria play an important role in the yak rumen. Previous studies have shown that Bacteroidota is mainly responsible for energy conversion and acquisition; while Firmicutes play an important role in the degradation of non-fibrous material (Evans et al., 2011; Reigstad and Kashyap, 2013; Ya-Bing et al., 2016). Among the two groups considered in this study, Bacteroidota was more abundant in the LC group, while Firmicutes were more abundant in the HC group, indicating that the number of bacteria involved in ruminal starch digestion and metabolism was significantly increased in yaks fed with high concentrate.

At the genus level, *Christensenellaceae_R-7_group*, *Prevotella* and *NK4A214_group* were the dominant bacteria in the rumen in this experiment. *Christensenellaceae_R-7_group* belongs to the phylum of thick-walled bacteria (Waters and Ley, 2019), which mainly catabolizes fibrous material (Evans et al., 2011). *Prevotella* is a protein-degrading bacterium of the rumen and gastrointestinal tract of ruminants; it mainly degrades the hemicellulose component of the food and promotes the degradation of non-fibrous polysaccharides and pectins (Purushe et al., 2010). In the present study, *Christensenellaceae_R-7_group* and *Prevotella* were more abundant in the LC group, which may be related to the low level of concentrate fed to the yaks. The *NK4A214_group* belongs to the family Rumenococcaceae, rumen bacteria are rich in endo-1, 4-beta-xylanase and Cellulase genes, these genes play an important role in the degradation of cellulose and hemicellulose, which are degraded to produce short-chain fatty acids that are available for use by the host. Thus, its relative abundance is related to the diet concentrate ratio (Koike and Kobayashi, 2009; Biddle et al., 2013). The relative abundance of NK4A214_group was found to be associated with isobutyl acid and isovaleric acid concentrations were positively correlated (Liu C. et al., 2019a). In the present study, *NK4A214_group* was more abundant in the HC group, similar to a previously report (Chen et al., 2021), this could explain the higher isobutyl acid and isovaleric acid concentration in the HC group. Some bacteria, including *Ruminococcus, Acetitomaculum*, *Candidatus_Saccharimonas* and *NK4A214_group*, were positively correlated with acetate, butyrate, isovalerate, propionate and isobutyrate concentrations, respectively, indicating that these bacteria may favor VFA production. For instance, *Ruminococcus* can ferment cellobiose or cellulose to produce butyric acid (Henderson et al., 2015). *Acetitomaculum*, which is mainly found in ruminants fed high concentrate diet, can produce acetic acid from monosaccharides (Hua et al., 2017). Due to the complex interactions between bacteria (Olotu et al., 2019), it is difficult for us to understand the bacterial activities that directly produce VFA (Mahowald et al., 2009).

Microbial flora has an important role in ecosystem function, and the relationships among microorganisms involved in ruminal fermentation ecosystems are very complex (Liu H. et al., 2019b). In this study, key microbial taxa with topological properties in the rumen of yaks were identified for the first time. The results showed that high concentrate diet altered the correlation between components of microbial flora. Network analysis can reveal interactions between species in both positive and negative ways (Liu et al., 2020). Negative interactions may weaken competitive relationships, while positive interactions may strengthen them back (Fan et al., 2018). In our study, compare to the low concentrate diet group, the high concentrate diet group revealed stronger negative correlations. We hypothesized that the cellulose-degrading bacteria in the rumen were inhibited due to the low cellulose content of the high concentrate feeds, which resulted in fewer bacteria. This further suggests that high concentrate rations can regulate the microbial dynamics of the rumen in yaks.

In the current study, we used PICRUSt 2 to predict the function of the ruminal microbial community in yaks. The data suggest that the assessment of gene functions of the ruminal microbiota is significantly influenced by the ration concentrate ratio. In most cases, genes involved in the metabolism of amino acids in the level 2 KEGG pathway were enhanced in yaks fed high concentrate diets. This suggests that the ruminal microflora of yaks fed high concentrates produce large amounts of protein, which provide the host with raw materials such as protein, which in turn sustains life and normal metabolism. It is worth noting that valine, leucine and isoleucine biosyntheses are the most expressed pathways, directly involved in amino acid metabolisms. In this study, valine, leucine and isoleucine biosyntheses were active at significantly higher levels in HC group, indicating that the increase in the percentage of concentrates increased the protein content of the rations, creating a more favorable environment for fermentation and the growth of cytolytic bacteria, which promote the participation of rumen microorganisms in the digestion and metabolism of nutrients. However, our results are based on predicted macrogenomics only and may not be representative of the actual function of ruminal bacteria. Further macrogenomic analyses are needed to explore the mechanism of these gene functions in feeding high concentrate diet to yaks.

## Conclusion

In this study, ruminal fermentation parameters and microflora of yaks that were fed high concentrate diets were analyzed. In fact, high concentrate diets altered the ruminal fermentation pattern, and the structure and composition of the microflora of yaks, which in turn affected their functions. The promotion of *Ruminococcus* and *Acetitomaculum* growth led to increased acetic acid and butyric acid contents in the rumen of yaks fed high concentrate diets, and the fermentation produced large amounts of VFA, tending to decrease ruminal pH and affecting yak ruminal health.

## Data availability statement

The datasets presented in this study can be found in online repositories. The names of the repository/repositories and accession number(s) can be found at: NCBI, PRJNA843719.

## Ethics statement

The animal study was reviewed and approved by Institutional Animal Care and Use Committee of the Qinghai University. Written informed consent was obtained from the owners for the participation of their animals in this study.

## Author contributions

SW, SC, and KP contributed to the conception and design of the study. KP, SW, and XW collected the samples. SW, KP, YY, HW, XB, and SC conducted relevant experiments. KP and SW organized the database. XW and SL performed the statistical analysis. KP wrote the first draft of the manuscript. XW, YY, SL, HW, and SC wrote sections of the manuscript. All authors contributed to the article and approved the submitted version.

## Funding

This research was funded by the National Key Research and Development Program of China (grant no. 2018YFD0502300), Qinghai Provincial Science and Technology Department of China (grant no. 2020-ZJ-935Q) and Qinghai ProviProvince’susands of High-end Innovative Talents Plan.

## Conflict of interest

The authors declare that the research was conducted in the absence of any commercial or financial relationships that could be construed as a potential conflict of interest.

## Publisher’s note

All claims expressed in this article are solely those of the authors and do not necessarily represent those of their affiliated organizations, or those of the publisher, the editors and the reviewers. Any product that may be evaluated in this article, or claim that may be made by its manufacturer, is not guaranteed or endorsed by the publisher.

## Footnotes

^1^ http://drive5.com/*uparse*/

^2^ https://gephi.org/
